# The Role of Mediterranean Diet and Intermittent Fasting in Modulating Inflammation and Clinical Biochemistry Markers in Hereditary Cancer Syndromes: A Review

**DOI:** 10.1007/s13668-025-00713-5

**Published:** 2025-12-03

**Authors:** Francesco Cacciabaudo, Giulia Accardi, Francesca Carta, Salvatore Tarantino, Giuseppe Annunziata, Tancredi Didier Bazan Russo, Paula Silva, Giuseppina Candore, Antonio Russo, Marcello Ciaccio

**Affiliations:** 1 Department of Biomedicine, Neurosciences and Advanced Diagnostics, Institute of Clinical Biochemistry, Clinical Molecular Medicine, and Clinical Laboratory Medicine, University of Palermo, Palermo, 90127 Italy; 2 Laboratory of Immunopathology and Immunosenescence, Department of Biomedicine, Neurosciences and Advanced Diagnostics, University of Palermo, Palermo, 90127 Italy; 3 Department of Precision Medicine in Medical, Surgical and Critical Care (Me.Pre.C.C.), University of Palermo, Palermo, 90127 Italy; 4 Department of Experimental Medicine, University of Campania “Luigi Vanvitelli”, Naples, Italy; 5 University of Porto (U. Porto), Porto, Portugal

**Keywords:** Hereditary cancer syndromes, Mediterranean diet, Intermittent fasting, BRCA, Inflammation, Biomarkers

## Abstract

**Purpose of review:**

This literature review of clinical and preclinical studies aimed to analyse the latest evidence on the Mediterranean diet (MD) and intermittent fasting (IF) to prevent or delay strategies for the onset of cancer in patients with hereditary cancer syndromes, correlating the data about some nutritional, clinical biochemical, and inflammatory biomarkers (e.g. CRP, IL-6, insulin, and IGF-1). Preliminary evidence from the literature suggests that MD and IF can favourably modulate markers of chronic inflammation and metabolism, potentially reducing the risk of cancer.

**Recent findings:**

Cancer risk increases significantly throughout life in patients with hereditary cancer syndromes such as BRCA1/2. Evidence suggests the role of lifestyle, especially nutrition, in delaying the onset of these diseases. Although existing studies point to promising roles for MD and IF in managing cancer risk, additional research is needed to clarify their long-term effects and define personalised guidelines for individuals with hereditary cancer syndromes.

**Summary:**

Preliminary data suggest that the MD and IF may help delay cancer onset in hereditary syndromes by modulating inflammation and metabolism. More studies are needed to confirm long-term benefits and guide personalized prevention.

## Introduction

In 2022, the number of new cancer diagnoses was 20 million with 9.7 million deaths globally. The occurrence of specific cancers varies between the sexes, but slight variations occur in both sexes, with 9.57 million new cases in men and 9.18 million in women globally in 2022 [1]. Cancer is a multifactorial chronic disease that is influenced by genetic predisposition and modifiable lifestyle factors, and diet and exercise play a significant role in cancer onset, prevention, and control. Hereditary cancer syndromes (HCSs) account for approximately 10% of cancer incidence, although currently underdiagnosed [2]. HCSs occur due to genetic germline mutations, with significant increases in cancer risk compared to sporadic cancer. Genetic alterations, the molecular basis of HCS, define the sequence changes in DNA that affect gene expression and protein function [3]. Hereditary breast cancer (HBC) and hereditary ovarian cancer (HOC) are the most frequent HCS in women, with the best-studied genetic variants in the BRCA1 and BRCA2 genes [4]. The lifetime risk of HBC is approximately 1 in 8 in Western countries, and HOC is much less common [5]. BRCA1 and BRCA2 are tumour suppressor genes that encode proteins involved in the double-strand break DNA repair mechanism by homologous recombination. Cells lacking these genes are more prone to accumulate errors in their DNA strands, resulting in a substantially increased risk of breast and ovarian cancers [6]. Despite the higher frequency of cancer in individuals with germline mutations, the HCS lifestyle can also influence age and severity of onset [7]. Over time, there has been a notable link between cancer prevention and the Mediterranean Diet (MD). This diet is a great source of bioactive molecules possessing anti-inflammatory, antioxidant, and most importantly, anticancer properties that have been well documented, such as polyphenols and polyunsaturated fatty acids. MD contributes to the prevention of various cancers, as demonstrated by several studies [8–12]. Intermittent fasting (IF) has also been shown to promote overall health and prevent chronic diseases, including cancer, through several metabolic mechanisms [13]. This dietetic protocol, composed of alternating periods of fasting and feeding, triggers profound metabolic changes, including decreased insulin levels and enhanced fat breakdown [14]. Biological mechanisms triggered by fasting, such as autophagy and sirtuin activation, were associated with anticancer effects [15, 16]. In addition, IF triggers a metabolic rewiring in cancer cells, increasing oxidative stress and treatment sensitivity [17–19]. Therefore, combining IF and MD may be a new approach in cancer prevention, even though further studies are needed to fully understand the mechanisms of this combination and its efficacy. Understanding how nutritional interventions affect biochemical and inflammatory markers is needed to explain the relationship between nutrition and HCS. In particular, circulating biomarkers such as C-reactive protein (CRP), interleukin-6 (IL-6), tumour necrosis factor-alpha (TNF-α), and other cytokines offer quantifiable information about the systemic inflammatory status. Metabolic biomarkers, including fasting glucose, insulin, and lipoproteins, can be used to track the impact of dietary interventions on insulin sensitivity and energy metabolism. Recent evidence has reported that diet modulation of such biochemical parameters may influence both cancer onset and overall metabolic well-being, highlighting the importance of incorporating biochemical profiling in experimental research and clinical management of individuals with HCSs [19, 20].

This paper reviews the literature on the effects of MD and IF on nutritional, inflammatory, and clinical biochemical markers in patients with HCS. It also outlines how these lifestyle interventions might affect cancer risk and overall health outcomes in genetically predisposed individuals, describing both the current evidence and areas where clinical studies are required.

In this context, MD and IF emerge as promising non-pharmacological strategies that may complement genetic counselling and clinical surveillance in hereditary cancer syndromes, although targeted evidence in HCS populations remains limited.

## HCS: Inflammation as a Key Modulator of Cancer Risk

Although familial clustering of colorectal, endometrial, breast, and ovarian cancers has been observed for a long time, the biomolecular mechanisms of inherited cancer susceptibility have only recently been elucidated. Among the first mutations associated with HCSs, MSH2 was associated with Lynch syndrome (LS). Subsequently, mutations were discovered in BRCA1 and BRCA2, which are not only responsible for hereditary breast and ovarian cancer but also for prostate and pancreatic cancer syndromes. Regarding hereditary breast and/or ovarian cancer syndromes, it is now known that the panel of genes involved is not limited to BRCA1 and BRCA2 but is a broad panel that includes genes with high and moderate penetration, such as TP53, PTEN, STK11, CDH1, PALB2, ATM, CHEK2, BARD1, RAD51C, RAD51D, and BRIP1 [21, 22]. As reported, mutations in specific genes lead to an increase in the development of some types of tumours, as the risk of breast cancer for BRCA1/2 mutation carriers is 60–80%, while that of ovarian cancer is 20–45%. Various preventive measures include prophylactic mastectomy, salpingo-oophorectomy, anti-oestrogen therapy, and increased imaging protocols [23]. It has also been shown that individuals with HCS often have chronic low-grade inflammation and metabolic dysregulation, which also influences cancer risk. Chronic inflammation is recognised as the basis of oncogenesis. [24, 25]. It has been highlighted that among BRCA mutation carriers, excess adiposity and dysmetabolism, and consequently, frequent increased concentrations of IGF-1, have been associated with a higher onset risk [26]. Unhealthy lifestyle habits amplify inflammation and combine with genetic vulnerability to increase cancer risk. However, lifestyle modification can counteract it. In particular, caloric restriction, weight control, and exercise have been shown to reduce cancer incidence, including in genetically vulnerable individuals [27–29]. In LS patients, early results suggest that an increase in high-fibre diet, a reduction in red meat consumption, and smoking quitting may reduce colorectal cancer risk [30, 31]. Therefore, lifestyle can significantly influence the onset of cancer, whether HCS or sporadic [32, 33].

In this multifaceted scenario, as inflammation is a complex process closely related to cancer, it is essential that oncological research moves away from the “cell-centric” vision of the tumour and instead amplifies its perspective, considering the totality of the systemic and, even more important, of the local network that together constitute the tumour microenvironment (TME). TME is composed of a huge number of immune mediators, both proteins and cells, such as T lymphocyte, neutrophils, macrophage, natural killer (NK) and NKT, inflammatory cytokines and chemokines, other than growth factors and proangiogenic mediators that can favour its growth and expansion [34, 35]. In some cases, chronic inflammation constitutes the background for tumour formation. Some causes of inflammation are localized and limited to an organ; others are systemic, such as all dysmetabolism conditions, which determine, as already highlighted, a chronic low-grade inflammation. Therefore, identifying modifiable risk factors, especially those that influence inflammation and metabolism becomes a strategic complement to genetic counselling, screening, and surveillance in patients with HCS [24, 36].

## Mediterranean Diet and Cancer Prevention

Overall, the MD model is a dietary pattern characterised by a particular abundance of bioactive components capable of exerting a protective effect against cancer. Several studies have examined the impact of typical Mediterranean foods or their components on reversing various hallmarks of cancer cells, such as regulation of proliferative signalling, genomic instability, immune destruction evasion, angiogenesis, metastasis, and apoptosis resistance, thus potentially targeting the hallmarks of cancer [37].

The MD, due to its features in terms of regular consumption of whole cereals, fruits, and vegetables, has been attributed to reduced risk of death and certain types of cancer reducing the inflammatory burden [20] (Table 1). Systemic inflammation and cancer risk can be controlled through lifestyle intervention, including the management of the diet, considering the quality and quantity of food and specific nutrients and nutraceuticals, as reported in literature [38, 39]. Interestingly, the MD also includes polyunsaturated fatty acids (PUFAs) from fresh fish, nuts, and seed oils that exert anti-inflammatory and antineoplastic effects by inducing autophagy and apoptosis in human cancer cells [11]. Other than omega-3 fatty acids, MD is also rich in polyphenols, such as isoflavones, phenolic acids, terpenes, and catechins, and monounsaturated fats, which, by suppression of oxidative stress and inhibition of the NF-kB signalling cascade, have an immunomodulatory effect, resulting in a reduction of pro-inflammatory cytokines levels, such as IL-6 and TNF-α, and in a possible antitumor activity [12, 40–42]. Among the main polyphenol-rich Mediterranean foods, extra virgin olive oil shows preventive activity due to its high concentrations of hydroxytyrosol, tyrosol, and secoiridoids oleuropein and oleocanthal [43]. MD has also been shown to result in a reduction of CRP and IGF-1 which, at high levels, induce a pro-tumour metabolic profile [44–46]. These molecules are also capable of modulating the activity of insulin, potentiating insulin resistance, which is a risk factor for most cancers [47]. The mechanisms through which the bioactive components of MD influence the tumorigenesis signalling pathways have been studied thoroughly both in vitro and in vivo [48] Specifically, natural molecules like resveratrol, retinoids, epigallocatechin gallate, and omega-3 PUFAs exhibit anticancer activities by inducing cell cycle arrest and pro-apoptotic pathway activation and by blocking proliferation and inflammation receptors and enzymes [9, 49]. Further research should examine the impact of strictly plant-food dietary patterns on cancer endpoints [50]. The main evidence on the effects of MD and bioactive components in its typical foods is summarised in Table 1.

**Table 1:** Cancer and mediterranean diet-associated effects. The table shows the main effects of MD on specific cancer types, according to literature

Cancer type	Main effects of MD
*CRC*	In CRC, high diet quality (like MD), particularly high intake of dietary fibre and calcium, is associated with a decreased risk. Adherence to the MD can reduce CRC risk by up to 45% [51].
*BrC*	In BrC, which doubles in incidence every 10 years until menopause, the Westernized lifestyle further increases the risk. High adherence to the MD, measured by adapted relative MD score (excluding alcohol), is associated with a 6% risk reduction, reaching 75% in postmenopausal women. When using alternative indexes such as arMD or MDS, the risk is reduced by 40% in postmenopausal women. The individual MD components, like fruits and vegetables, PUFA n-3, olive oil, and others, show promising effects on protection from BrC [52–55].
*PC*	In PC, diets high in animal-derived fats are associated with increased incidence, while a greater intake of fibre, green tea, and protective nutrients (e.g., folates, lycopene, and vitamin C) appears to reduce the risk [56].
*BC*	For BC, dietary factors are critical, as many substances are excreted through the urinary tract. Olive oil, in particular, has been shown to exert a protective effect [57].
*CC*	In CC, folates and vitamins from fruits and vegetables may protect DNA from damage. The MD is also associated with a lower risk of high-risk HPV infection, slowing its progression and contributing to a 60% reduction in CC incidence [58].
*EC*	In EC, excess endogenous or exogenous oestrogens increase DNA replication and the likelihood of mutations, especially when unopposed by progesterone, enhancing mitotic activity. Moderate adherence to the MD reduces risk by 43%, and high adherence by 49% [59–61].
BTPC	For BTPC, greater adherence to the MD (measured by modified MDS) is inversely associated with gallbladder cancer risk. Although evidence is limited for pancreatic cancer, a healthy lifestyle and MD adherence appear to reduce the risk [62, 63].
*LC*	In LC, particularly among heavy smokers, increased adherence to the MD may reduce the risk by 62% [64].
*HNC*	For HNC, high fruit and vegetable intake offers protection, especially against oral cavity cancers [65].
*GC*	In GC, although Helicobacter pylori is a major confounder, greater adherence to the MD is associated with a 22–43% reduction in risk [66].

Abbreviations: *CRC* colorectal cancer, *MD* mediterranean diet, *BrC* breast cancer, *arMD* adapted relative mediterranean diet, *MDS* mediterranean diet score, *PUFA* polyunsaturated fatty acids, *PC* prostate cancer, *BC* bladder cancer, *CC* cervical cancer, *EC* endometrial cancer, *HPV* human papillomavirus, *BTPC* biliary tract and pancreatic cancer, *LC* lung cancer, *HNC* head and neck cancer, *GC* gastric cancer

## IF and Cancer Prevention

**Fig. 1:**
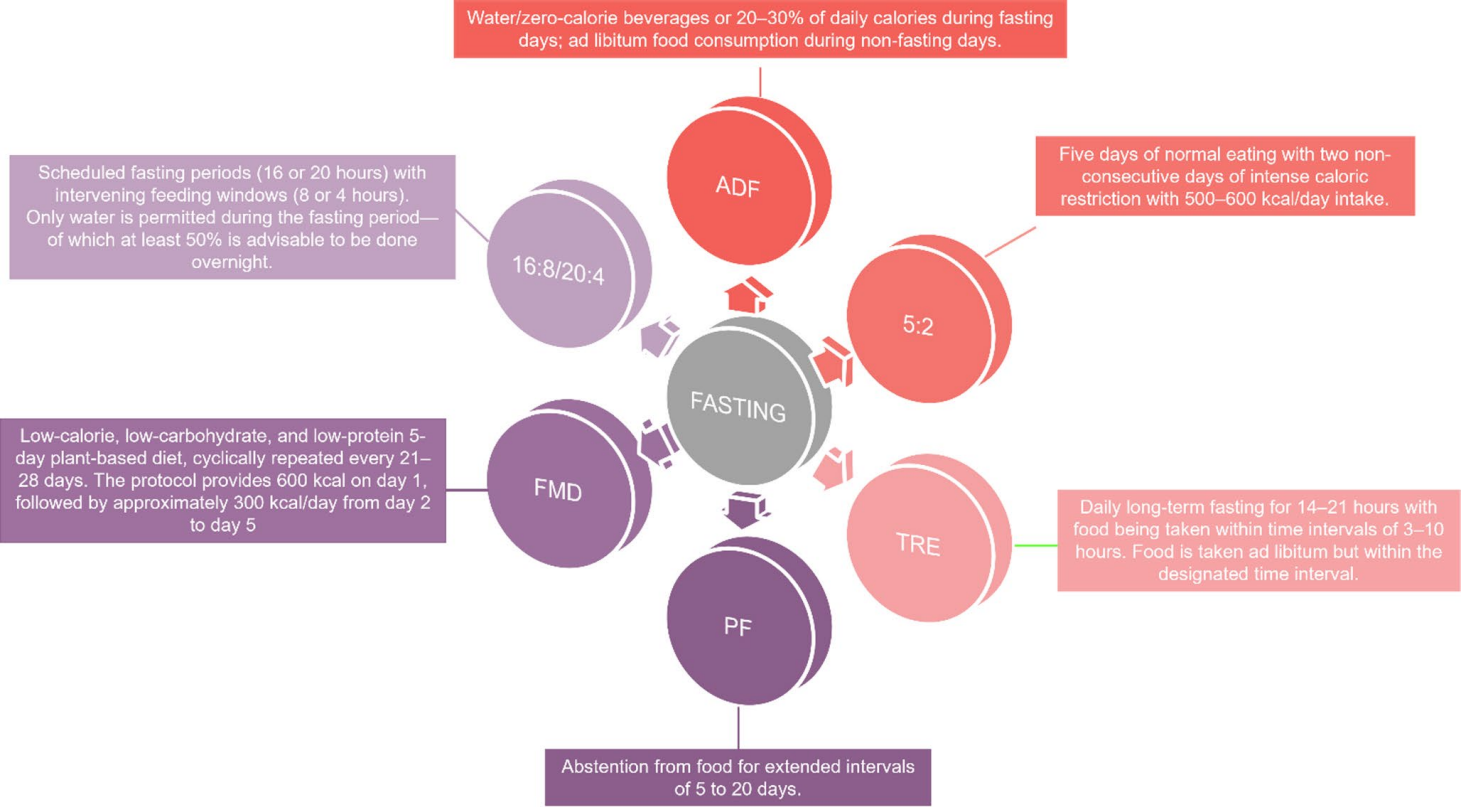
Different types of fasting

Fasting is defined as a voluntary abstinence from all foods or specific food types. Various forms of fasting have been practiced for thousands of years by different religions, each with its own beliefs and norms [67] (Fig. 1). Short periods of fasting, where food is avoided, or caloric intake is significantly limited, are alternated with periods of unrestricted eating, focusing primarily on when rather than what to eat [68]. Standard IF protocols include alternate-day fasting, the 16:8 method, prolonged fasting, and weekly whole-day fasting. Some methods allow for 25% of daily caloric intake on fasting days, with no restrictions on feeding days [13]. During fasting, adequate intake of non-caloric fluids is often recommended to maintain hydration and physiological balance [14]. Preclinical and clinical studies on short-term fasting highlight key metabolic adaptations that may underlie IF’s health benefits [69, 70]. Fasting windows typically range from 16 to 36 h, though most do not exceed 24 h of complete caloric abstinence [67, 71]. These periods trigger distinct physiological responses compared to continuous caloric restriction, including improved insulin sensitivity, reduced oxidative stress, and shifts in lipid metabolism [70]. IF is, thus, considered a sustainable alternative to daily energy restriction for improving body composition and metabolic health [72, 73]. IF has been linked, by laboratory and epidemiologic studies, to enhanced systemic function and the prevention of obesity, improvement in body composition, specifically reduction in fat mass with minimal impact on lean mass, and improvement in metabolic parameters, including glycemia, lipid profile, and blood pressure [68]. These benefits arise from shifts in metabolic pathways as the body transitions to using adipose tissue and, to a lesser extent, muscle for energy and metabolite production [17]. Fasting induces metabolic stress, lowering insulin and increasing glucagon, promoting glycogenolysis and lipolysis [14]. Protocols like time-restricted feeding and alternate-day fasting enhance metabolic flexibility and autophagy, reducing cellular damage and malignancy risk. By lowering IGF-1 and insulin, IF modulates the inflammatory tumour microenvironment. Several studies, including clinical investigations, have shown that IF reduces IL-6, CRP, and TNF-α levels and suppresses IGF-1, which can limit unrestricted cell growth. In the long term, this maintains many organs: brain, muscle, liver, and adipose tissue, through adaptive biological mechanisms [67]. The brain uses ketone bodies increasingly for energy, while peripheral tissues utilize mainly fatty acids [74]. Ketogenesis stimulates gluconeogenesis to maintain plasma glucose at around 70 mg/dL to supply the brain constantly. Ketone bodies are histone deacetylase inhibitors, a reaction that can decelerate tumour growth [75]. β-Hydroxybutyrate is an endogenous histone deacetylase inhibitor that resists oxidative stress [76]. Glucocorticoids and adrenaline are among the hormones that maintain glycaemic and increase lipolysis during fasting [77]. IF also increases FGF21, which downregulates IGF-1 via inhibition of the phosphorylation of hepatic STAT5. Concurrently, IGFBP1 binds to circulating IGF-1, limiting its receptor activation and bioactivity [78]. Thus, downregulation of IGF-1 increases tolerance to chemotherapy and reduces its side effects, as demonstrated in murine models [79]. Elevated IGF-1 has been implicated in the development of colon, prostate, and breast cancers through its anti-apoptotic and pro-proliferative effects [80]. Fasting lowers glucose, IGF-1, insulin, and leptin levels and raises adiponectin levels by regulating the IGF-1/mTOR pathway. These activities result in IF antitumour properties by suppressing free radical formation and enhancing stress resistance. Reduced IGF-1 and insulin levels may protect normal cells from anticancer treatments [79]. Autophagy and sirtuin activation caused by fasting assist in cellular repair and tumour suppression [60]. Autophagy disrupts defective cellular components and promotes genomic stability [16]. It supports cellular homeostasis, degrades mutagenic agents, fights oncogenic infections, and preserves stem cell functions. In addition, it enhances chemotherapy efficacy, also contributing to treatment resistance [81, 82]. By reducing glucose and increasing fatty acid oxidation, fasting shifts cancer cell metabolism from aerobic glycolysis to mitochondrial respiration, thereby increasing ROS levels. This leads to oxidative stress and impairs redox buffering owing to reduced glutathione synthesis, which enhances chemotherapy effectiveness [19, 79]. Compared with caloric restriction, IF may be more sustainable over time. Most of the data on its anticancer potential have been derived from preclinical models. In mice, fasting is limited to 48–60 h, beyond which starvation risk increases, whereas humans can fast for weeks [83]. The current study explored IF’s role of IF in cancer prevention and progression. While IF reduces body weight and influences tumour biology, clinical trials assessing its effects on hormonal and inflammatory cancer markers remain inconclusive. Evidence of its impact on cancer incidence and prognosis in humans remains limited due to the lack of long-term studies. Despite these gaps, IF stands out for its low cost, minimal side effects, and compatibility with healthy lifestyle interventions, making it a promising candidate for oncology research [83]. IF has also attracted attention for its potential role in HCS prevention [84].

This figure summarises the main nutritional approaches that share fasting as a common feature. There are two types of ADF: (1) Zero-calorie ADF, where only water or zero-calorie beverages are allowed during fasting days, and (2) Modified ADF, in which 20–30% of the regular caloric intake is consumed during fasting days. Food was consumed ad libitum on non-fasting days [85]. The 5:2 diet has five days of normal eating with two non-consecutive days of intense caloric restriction with 500–600 kcal/day intake [85]. TRE is a daily long-term (14–21 h) fasting with food consumed within time intervals of 3–10 h. Food is consumed ad libitum but within a designated time interval [85]. PF involves abstention from food for extended intervals of 5–20 d. Thisprotocol induces a metabolic shift known as the ‘metabolic switch’, in which the body transitions from using glucose to ketone bodies as the primary source of energy. This is typically converted after 12–36 h of starvation, and ketone levels (e.g. beta-hydroxybutyrate and acetoacetate) increase between days 5 and 10. Several beneficial changes have been observed, including weight reduction (2–10%), reduction in waistline, and lowering of systolic and diastolic blood pressure. However, several side effects have also been observed —presumably concerning the length of the fast—namely, insomnia, muscle wasting, decreased levels of HDL cholesterol, tiredness, and hunger [86]. FMD consists of a low-calorie, low-carbohydrate, and low-protein 5-day diet that is cyclically repeated every 21–28 days. It consists of a plant-based diet designed to induce metabolic changes similar to those induced by complete fasting, but with greater safety and compliance. The protocol provided 600 kcal on day 1, followed by approximately 300 kcal/day from days 2 to 5. FMD is intended to reduce glucose, insulin, and IGF-1 (which can promote tumour growth) levels but induce ketosis and fasting-like metabolic effects [87]. IF involves scheduled fasting periods (usually 16–20 h) with intervening feeding windows (8–4 h). Under the 16:8 protocol, 16 consecutive hours of total caloric abstinence were completed using an 8-hour eating window. Only water is permitted during the fasting period, of which at least 50% is advisable to be provided overnight (i.e. 8 h at night). In the remaining 8 h, food may be consumed normally. The 20:4 protocol consisted of 20 consecutive hours of caloric fasting followed by a 4-hour eating window. No food or caloric beverages were consumed during the fasting period, but water was allowed. Similar to the 16:8 protocol, it is recommended to schedule approximately 50% of the fasting time at night [88]. Abbreviations: alternate-day fasting, ADF; time-restricted eating, TRE; prolonged fasting, PF; fasting-mimicking diet, FMD; intermittent fasting, IF.

## Combined Effects of MD and IF

**Fig. 2:**
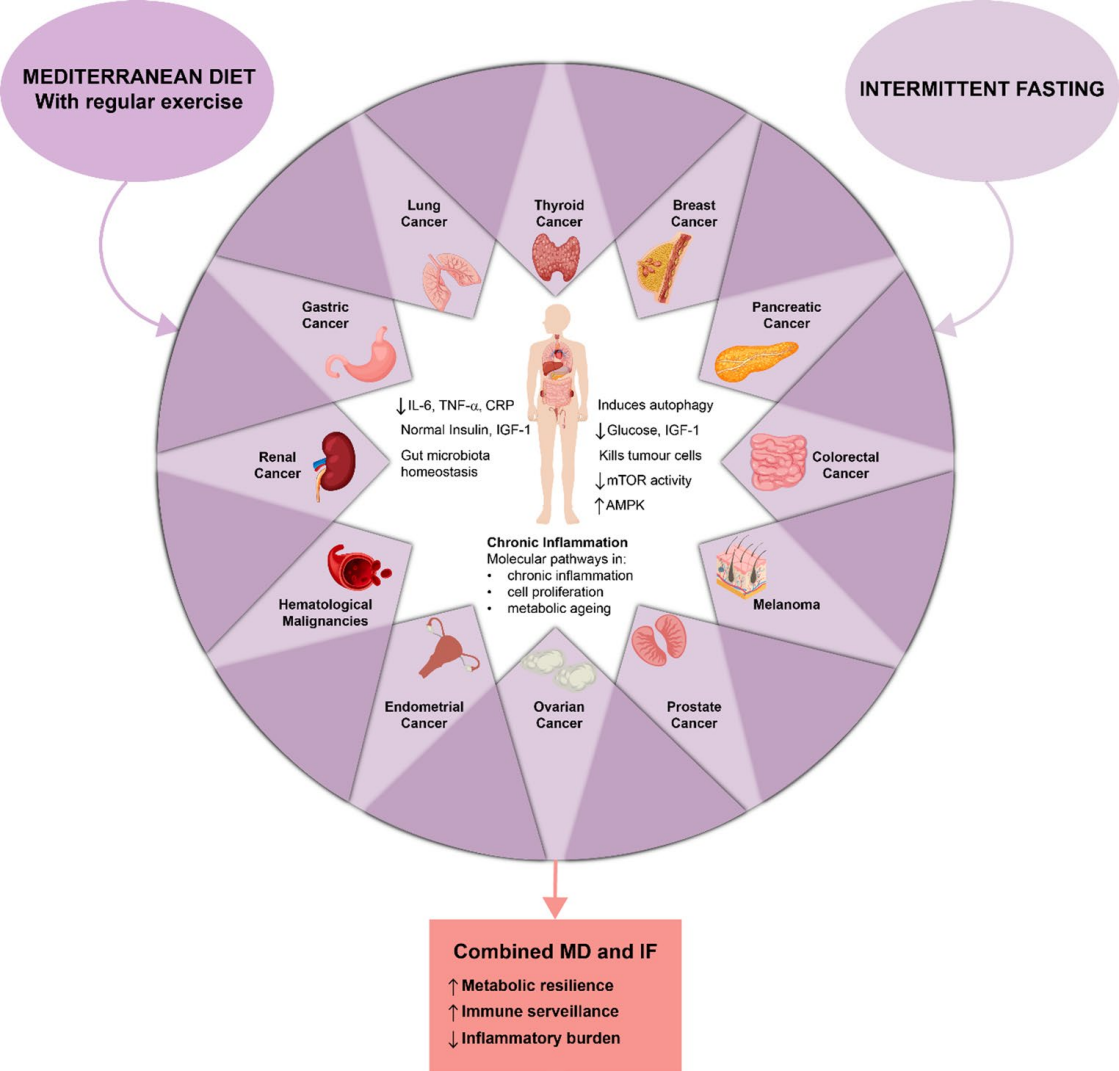
Synergistic effects of the Mediterranean diet (MD) and intermittent fasting (IF) on chronic inflammation and cancer prevention

Many studies have reported the beneficial effects of MD and IF on metabolic function and cancer risk. However, few studies have addressed the combination of these two approaches. The exact literature investigating the relationship between MD and IF in a genetically predisposed cancer population, such as the BRCA mutation-carrier group, remains limited. Longitudinal studies have not yet assessed the combined effects of risk biomarkers, clinical outcomes, and molecular parameters in high-risk populations. The current literature has several limitations, including protocol heterogeneity due to the use of different variants of fasting (16:8, 5:2, alternate-day fasting -ADF, etc.), making standardisation and comparison across studies difficult; limited study duration, typically 4–12 weeks, too brief to measure effects on carcinogenesis; limited populations studied, with most evidence in healthy subjects or individuals with obesity and few data points for patients with cancer or genetic mutation carriers; problems of adherence and compliance since maintaining both methods over the long term is difficult and dropout can cause bias; and environmental confounders such as lifestyle and exercise, which can obscure the specific effects of dietary interventions. These limitations underscore the need for long-term randomised controlled trials with clinically or molecularly meaningful oncological endpoints. Both approaches address the molecular pathways involved in chronic inflammation, cell proliferation, and metabolic ageing. In addition to short duration and protocol heterogeneity, external validity for HCS carriers remains limited, as most trials enrolled healthy, overweight/obese, or oncology cohorts without germline-mutation stratification. Hence, extrapolations to BRCA1/2 and other HCS populations should be considered provisional until HCS-specific trials are available. As demonstrated by preclinical and clinical studies reviewed in the literature, MD, especially when accompanied by regular exercise, can reverse low-grade chronic inflammation, which is a recognised factor in the initiation and promotion of cancer and its growth. Owing to its anti-inflammatory and antioxidant profile, MD decreases inflammatory markers such as IL-6, TNF-α, and CRP, normalises insulin and IGF-1 concentrations (both of which are involved in cancer formation), and normalises gut microbiota homeostasis by increasing the integrity of the intestinal barrier [89]. On the other hand, IF activates autophagy, a key cellular process involved in the breakdown and recycling of damaged components, thereby suppressing tumour growth. IF has been shown to induce autophagy, reduces.

blood glucose and IGF-1 levels, induces apoptosis and oxidative stress, kills tumour cells while sparing normal cells, and enhances tolerance to cancer therapy. mTOR and AMPK are key pathways involved in fasting-induced increases in ROS and mitochondrial stress [90]. Model data, such as Ramadan fasting (12–14 h per day for 30 days), show induction of autophagy under nutritional stress with beneficial effects on longevity, stress resistance, and protection against chronic diseases. Fasting is associated with decreased mTOR activity, low insulin and IGF-1 levels, augmented immune response, cellular longevity, and suppressed inflammation; this finding is based on a clinical study [91]. There is also encouraging evidence, supported by clinical trials, of synergistic action when MD is used in conjunction with IF, where MD provides protective nutrients during the fed state. IF amplifies the effects of intermittent caloric restriction. No trials have yet tested the combination in oncologic or genetically predisposed patients, although there are some data in other disease states. In an ADF clinical trial, treatment with IF of 36 h of daily fasting interspersed with 12 h of ad libitum intake led to spontaneous weekly caloric restriction of ~ 37% accompanied by weight loss, increase in the fat-to-lean mass ratio, reductions in abdominal fat, T3 levels, heart rate, and blood pressure, and a decrease in Framingham Risk Score. Within six months, ADF also lowered cholesterol, LDL, VLDL, triglycerides, and inflammatory biomarkers such as sICAM-1, along with alterations at the molecular level, such as increased β-hydroxybutyrate and PUFA levels, and conversion of energy to lipid metabolism pathways [92]. Additional evidence suggests that IF and caloric restriction control the mTORC1 pathway, which affects cell growth, survival, and autophagy. IF also activates AMPK and CREB, with downstream effects on circadian control and metabolic flexibility [93]. Such a synergistic action offers a promising method for managing inflammation and IGF-1 signalling in patients with hereditary cancer syndromes. The combination of MD and IF may increase metabolic resilience, improve immune monitoring, and reduce the inflammatory load, potentially preventing or delaying cancer initiation in susceptible individuals. Importantly, no clinical trials have specifically investigated the combined effects of MD and IF in carriers of HCS. Current evidence derives almost entirely from preclinical studies and non-HCS clinical cohorts; therefore, extrapolations to genetically predisposed populations should be interpreted with caution. Further clinical trials are required to clarify the molecular mechanisms underlying these effects and to create individualised dietary recommendations based on genetically susceptible populations. For an overview of the effects of MD and IF, see Fig. 2.

This diagram illustrates how MD and IF modulate key molecular pathways involved in chronic inflammation, cell proliferation, and metabolic ageing. The Mediterranean diet, particularly when combined with regular physical activity, reduces inflammatory markers (interleukin-6 [IL-6], tumour necrosis factor alpha [TNF-α], and C-reactive protein [CRP]), normalises insulin and insulin-like growth factor 1 (IGF-1) levels, and promotes gut microbiota homeostasis. IF enhances autophagy, lowers blood glucose and IGF-1 levels, and induces selective tumour cell death while preserving healthy cells, partly through modulation of the mammalian target of rapamycin (mTOR) and AMP-activated protein kinase (AMPK) pathways. The combined use of MD and IF may enhance metabolic resilience, improve immune surveillance, and reduce inflammatory burden, offering a promising strategy for preventing or delaying cancer in at-risk individuals.

## Influence of Inflammatory Biomarkers in Hereditary Cancer-Predisposed Patients: the Impact of Mediterranean Diet andIF

Chronic inflammation is a key characteristic of cancer and is involved in the initiation, promotion, and progression of tumours [94]. The inflammatory microenvironment can also fuel oncogenic pathways in HCS carriers, such as BRCA1/2, TP53, or MLH1 mutations [95]. Increased circulating concentrations of inflammatory markers (for example CRP, IL-6, TNF-α, and high-sensitivity IL-1β) have been observed in HCS, reflecting synergistic relationships between genetic predisposition and systemic inflammation [26]. Concurrently, alterations in glucose and lipid metabolism generally occur in these disorders. The IGF-1 axis, for instance, illustrates differential regulation in high-risk individuals [96]. Hypersecretion of IGF-1 is linked to augmented cell proliferation and reduced apoptosis, partially mediated by crosstalk with inflammatory networks such as NF-κB [97]. Within this framework, nutritional intervention offers an encouraging strategy to reduce the risk of onset. MD has also often been associated with lower levels of CRP and IL-6 and increased insulin sensitivity, according to clinical trials [98, 99]. IF also modulates metabolic and inflammatory parameters in another way by lowering insulin levels, activating adiponectin secretion, and influencing leptin signalling in a beneficial way [14]. In individuals with HCS, these diets lower inflammation systemically and can control oncogenic pathways and their activators, including IGF-1 and proinflammatory cytokines [67]. Longitudinal measurements of these biomarkers and their respective metabolic markers may explain the extent to which MD and IF interventions effectively decrease cancer risk among genetically susceptible individuals and guide targeted preventive interventions [100]. See Table 2 for further details.

**Table 2:** Biomarker changes induced by MD/IF: Evidence overview

Biomarker	Modulation by MD/IF	Evidence level	Reference
CRP IL-6	Reduced by MD	Clinical	[98]
Glycemia Insulin	Reduced by MD	Preclinical	[99]
Insulin Leptin	Reduced by IF	Preclinical	[14]
CRP Glycemia IGF-1 Body Weight Abdominal Fat	Reduced by IF	Preclinical and clinical	[67, 100]

## Conclusion

The existing literature clearly supports the protective role of MD and IF in limiting inflammation and possibly lowering the risk of cancer in patients with HCSs such as BRCA1/2 mutations. Considering that current human evidence largely comes from non-HCS populations, multicentric, well-designed longitudinal trials are necessary to confirm these findings and set the stage for tailoring nutritional advice in inherited cancer prevention. Future research should prioritise biomarker-guided interventional trials specifically in BRCA1/2 carriers and other HCS cohorts, with appropriate stratification by sex and rigorous monitoring of dietary adherence. Understanding how lifestyle changes can influence genetic risk could lead to new targets for integrative approaches to cancer prevention and treatment while also clarifying which hypotheses remain speculative and which are supported by robust clinical evidence. This would underscore the ultimate goal of improving the quality and outcome of life in high-risk individuals.

## Key References

- Liu J, Zhang R, Ma L, Yang P, Wu Z, Chen Y, Peng J, Yang X, Huang C, Yan J. Association of dietary carbohydrate ratio, caloric restriction, and genetic factors with breast cancer risk in a cohort study. Sci Rep. 2025 Feb 20;15(1):6263. https://doi.org/10.1038/s41598-025-90844-0
  - ○ The selected paper is the first large prospective study to integrate detailed dietary data with germ-line variants for cancer predisposition, demonstrating that lower carbohydrate intake and mild caloric restriction synergise with protective alleles to reduce breast-cancer incidence. It provides pivotal evidence that nutrition can modify cancer‐predisposition penetrance.
- Sessa C, Balmaña J, Bober SL, Cardoso MJ, Colombo N, Curigliano G, Domchek SM, Evans DG, Fischerova D, Harbeck N, Kuhl C, Lemley B, Levy-Lahad E, Lambertini M, Ledermann JA, Loibl S, Phillips KA, Paluch-Shimon S; ESMO Guidelines Committee. Electronic address: clinicalguidelines@esmo.org. Risk reduction and screening of cancer in hereditary breast-ovarian cancer syndromes: ESMO Clinical Practice Guideline. Ann Oncol. 2023 Jan;34(1):33-47. https://doi.org/10.1016/j.annonc.2022.10.004
  - ○ The selected paper offers the most up-to-date, consensus-driven recommendations for managing BRCA-associated cancer risk; notably emphasises lifestyle interventions (dietary patterns, weight control, timed eating) as adjuncts to surgery and chemoprevention, underlining their clinical relevance.
- Bagheri A, Asoudeh F, Rezaei S, Babaei M, Esmaillzadeh A. The Effect of Mediterranean Diet on Body Composition, Inflammatory Factors, and Nutritional Status in Patients with Cachexia Induced by Colorectal Cancer: A Randomized Clinical Trial. Integr Cancer Ther. 2023 Jan-Dec;22:15347354231195322. https://doi.org/10.1177/15347354231195322
  - ○ The paper presents the results of a randomised trial showing that a strict Mediterranean-diet protocol lowers CRP and IL-6 and improves lean-mass retention in oncology patients, reinforcing the idea that diet’s anti-inflammatory and metabolic benefits translate directly within a cancer setting.

## Data Availability

No datasets were generated or analysed during the current study.

## Abbreviations

HCS: Hereditary cancer syndrome
HBC: Hereditary breast cancer
HOC: Hereditary ovarian cancer
MD: Mediterranean diet
IF: Intermittent fasting
CRP: C-reactive protein
IL: Interleukin
TNF-α: Tumor necrosis factor-alpha
LS: Lynch syndrome
TME: Tumor microenvironment
NK: Natural killer
